# Imprinted habitat selection varies across dispersal phases in a raptor species

**DOI:** 10.1038/s41598-024-75815-1

**Published:** 2024-11-04

**Authors:** Florian Orgeret, Urs G. Kormann, Benedetta Catitti, Stephanie Witczak, Valentijn S. van Bergen, Patrick Scherler, Martin U. Grüebler

**Affiliations:** Swiss Ornithological Institute, Sempach, Switzerland

**Keywords:** Movement ecology, Early-life, Prospection, GPS telemetry, Conditional logistic regression, Individual specialization, Behavioural ecology, Biogeography, Ecological modelling, Evolutionary ecology, Population dynamics, Urban ecology

## Abstract

Natal Habitat Preference Induction (NHPI) plays a significant role in shaping settlement decisions in dispersive animals. Despite its importance, limited research has explored how NHPI varies during natal dispersal phases and across different types of natal habitats. In this study, we examined NHPI in 77 GPS-tagged juvenile red kites (*Milvus milvus*) originating from different natal habitats along an elevational gradient in Switzerland. We applied individual-based step selection analysis to investigate habitat selection from independence to settlement. We found that during the prospecting phase, individuals predominantly selected habitats similar to their natal environments. However, this pattern changed in the settlement phase: individuals fledged from habitats at higher elevations or closer to urban areas mostly avoided similar habitats (negative NHPI), while those from areas with more farmlands or pastures (combined with forests) showed a preference for similar habitats (positive NHPI). Moreover, the magnitude and individual variation in NHPI differed depending on the natal habitat types from which individuals originated. These findings highlight that strength, direction, and individual variation in NHPI differ between natal habitat types and dispersal phases. Natal habitats therefore can have pervasive legacy effects on subsequent habitat selection, likely affecting population and range dynamics.

**Supplementary Information:**

The online version contains supplementary material available at 10.1038/s41598-024-75815-1.

## Introduction

Natal dispersal consists of three phases: departure from the birth location, prospecting for suitable habitats, and final settlement at a breeding site^1–3^. Natal dispersal is central in shaping individual life-histories^4^ and intersects with developmental and behavioural factors affecting ecological and evolutionary outcomes^5–7^. Early-life experiences, particularly through mechanisms like ‘ecological imprinting’, have long been recognized to play a critical role in shaping dispersal patterns^8^. Imprinting also represents an important mechanism shaping individual preferences in food, habitat, mate, or host selection (reviewed in^9^), extending its impact beyond individual ecology to broader evolutionary processes, such as reproductive isolation and speciation^5,10,11^.

The concept of Natal Habitat Preference Induction (NHPI) generalizes processes of habitat imprinting and describes how early experiences in the natal habitat influence the likelihood of selecting a similar environment during later life-history stages^12^. Experimental support for NHPI has been found across a range of animal taxa, primarily in controlled laboratory conditions (reviewed in^12^, but see two studies that haven’t found any effects^13,14^). In free-living vertebrates, the influence of natal habitat on subsequent settlement habitat preferences has been demonstrated in mammals such as rodents^15–18^, canids^19–22^, and ungulates^23,24^, as well as in reptiles^25^ and fishes^13^. More recent studies have also strengthened the evidence for NHPI in birds^26–32^.

While the occurrence of NHPI is widely recognized, there is a gap in understanding its intraspecific variation. This variation includes individual differences, changes across life stages, and spatial differences related to natal habitat characteristics^12^. Examining this type of variation, especially across dispersal phases, is vital for comprehending population distribution and structuring, as well as habitat connectivity. Such insights have implications for habitat management and conservation strategies^33^, and for understanding demographic and evolutionary impacts^12,34^. Nonetheless, accurately quantifying NHPI variation is challenging. This is largely due to the complexities involved in monitoring the continuous habitat choices of animals in the wild, particularly during their natal dispersal, which is one of the most mobile phase in the life cycle of many species^35^.

Unlike adults, who are familiar with their surroundings, young animals are encountering their habitats for the first time and need to gather information. Their primary needs prior to breeding should be to ensure survival; therefore, they are expected to be less influenced by NHPI, as recently supported in a study on common loons (*Gavia immer*^29^). Juvenile loons preferentially visit lakes with pH levels similar to their natal habitats, choosing large lakes regardless of the size of their origin, which suggests they primarily seek to meet their immediate energetic needs for growth and migration preparation^29^. In contrast, young adults are strongly selecting lakes both of similar pH and size to their natal lake^26^. In juvenile white-tailed deer (*Odocoileus virginianus*^24^), NHPI was not apparent during mate searching excursions from the natal home range before departure. However, NHPI became evident after departure during the transience phase of dispersal, which included prospecting and settlement^24^. These studies underscore that the influence of NHPI can vary across life stages. However, the extent of this variation and its implications remain largely unexplored, particularly when comparing NHPI effects across different habitat types.

According to habitat selection theory, the intensity of NHPI should be influenced by the characteristics of the natal habitats from which individuals originate^34,36^. For instance, adverse conditions in specific habitat types could alter NHPI, potentially leading to its reversal, although instances of negative NHPI are expected to be rare^34^ (to our knowledge, only one study showed negative NHPI^21^). Consequently, individuals from a given population but originating from different habitat types might exhibit notable differences in NHPI^15,37^. Despite the potential for significant individual variation in habitat preferences later in life^32,34^, comparisons of NHPI effects across different natal habitats have rarely been conducted. To the best of our knowledge, only two studies have explored this topic^17,27^.

In habitat selection modelling, the focus typically lies on analysing population averages rather than on individual variation^38^. This is crucial because weak or null average effects in habitat selection could obscure significant individual preferences, particularly when there is substantial variation within or between individuals^39^. For example, individual members of a population might exhibit strong negative or positive habitat preferences based on their natal habitat types. However, these distinct preferences might not be evident in the overall population average, which could equal zero. This would suggest no apparent trend in habitat preference at the population level, despite significant variations among individuals. Thus, when investigating NHPI, it is crucial to differentiate between average effects and varying degrees of individual heterogeneity^39^. Therefore, a specific emphasis on assessing individual variation is essential in habitat selection modelling when investigating NHPI^17^, but is mostly neglected.

In this study, we examined NHPI in red kites (*Milvus milvus*) from different habitat types in Switzerland during both the prospecting and the settlement phases of natal dispersal. First, we conducted a combination of multivariate and cluster analyses to categorize natal habitats into distinct groups allowing for the investigation of differences in NHPI in relation to natal habitat types. We then applied individual-based integrated step selection analysis (iSSA^40,41^) to a tracking dataset of young red kites monitored for up to six years. By analysing individual movement trajectories during dispersal phases, we assessed the degree of dissimilarity between the habitats used and their respective natal habitats^24^ while considering individual variation in habitat selection coefficients^39^. Given that prospecting typically involves exploratory movements to sample potential settlement sites^1–3^, we expected habitat selection during the prospecting phase to be at least partially based on similarity to natal habitats, albeit to a lesser degree than during settlement, where we expected more pronounced habitat selectivity^42^. However, we also expected that this shift between dispersal stages is influenced by the conditions experienced during early life, which should vary with the natal habitat types^34^.

## Methods

All data preparation and analyses were done with the R software (R 4.3.3, R Core Team 2024).

### Study species

The red kite is a generalist raptor and opportunistic open farmland forager that hunts mainly on small mammals, bird, reptiles and amphibians, scavenges on carcasses, and also frequents semi-urban areas, feeding on roadkill, and human refuse such as slaughter waste and food scraps^43,44^. It exhibits generalist habitat use and can occur across a broad elevation gradient^42,45^. The species primarily breeds on the edge of forest patches within diverse agricultural landscapes, usually at medium to low elevations^45–48^. During natal dispersal, red kites alter their habitat selection. While they use a wide array of habitats and elevations during the prospecting phase, they show more specific preferences during the settlement phase^42^. They often avoid high elevations and steep slopes, opting for forest patches with medium forest density and areas proximate to their natal territories (typically within 50 km)^42^.

### Study area and tagging

Our study was conducted in western Switzerland (Cantons of Bern and Fribourg) over a 400 km^2^ area from 2015 to 2020. The region spans from the lowlands of the Swiss plateau to more mountainous terrain extending towards the Swiss Alps. The study area is characterized by a diverse range of habitats distributed along an elevation gradient: from low to medium elevations, it features arable lands interspersed with small cities and villages, as well as managed forests and large grassland areas^49,50^. At higher elevations, the landscape shifts to even more heterogeneous agricultural habitats, marked by pastures associated with more complex and heterogenous cultivation patterns (mosaic of small, cultivated land parcels with different cultivation types, scattered trees associated with houses or gardens). This diverse range of habitats available to red kites likely accounts for the high breeding density observed in the study area compared to other regions (approximately 130 breeding pairs per year^49^). At higher elevations, breeding has been shown to start later in the season^50^.

We analysed data from 78 nestlings originating from nests located along an elevation gradient ranging from 532 m to 1084 m. The birds were captured at their nest and brought to the ground for tagging. Nestlings were equipped with solar-powered GPS-GSM-UHF transmitters (either Ecotone SKUA/CREX or Milsar M9) and were monitored for up to six years. Transmitters were attached at approximately 40 days of age using a backpack-style diagonal-loop^50–52^. Following^50^, the weight of the transmitters (including the harness) was 26 g, which corresponds to 2.2–4.0% of the body weight at tagging (minimum of 670 g). All procedures involving the handling of birds were performed in strict accordance with ethical standards. The experimental protocols were approved by the Amt für Lebensmittelsicherheit und Veterinärwesen (LSVW) of the Canton of Fribourg (Permit No. 2017_29_FR) and the Federal Office for the Environment (FOEN). All methods were carried out in accordance with the relevant regulations and guidelines (ARRIVE guidelines https://arriveguidelines.org). Data was transmitted via GSM (with a spatially accuracy varying between 1 and 5 m) and stored on the Movebank data repository (movebank.org). The Ecotone transmitters recorded positions at 1-hour intervals, while Milsar tags recorded at 10-minute intervals. To make the tracks comparable, we resampled them into a continuous series of 1-hour movements for each individual, using the *amt* package^53^. Erroneous locations were filtered by a speed filter of 35 m/s^42^. One individual’s data had excessive gaps between locations and was subsequently removed from the analysis.

### Assigning natal territories to natal habitat types

To characterize the habitats within the natal territories of the studied species, we extracted habitat variables within a 2 km buffer radius surrounding each nest. This buffer size reflects the standard territory size for red kites in our study population^50^. Based on previous work on red kites^47,48^ we characterized the natal habitat using elevation and the proportions of Corine Land Cover (CLC) classes (spatial resolution of 100 m calculated over one year, see details in^42^): arable lands, grass lands, forest, urban (including cities and villages associated with the transport network), water (including lakes and rivers) and a remaining class that mainly corresponds to a mosaic of cultivation patterns in the study areas. Subsequently, we applied a combination of Principal Component Analysis (PCA) and Hierarchical Clustering on these variables from the natal areas to categorize them into distinct natal habitat type, using the *FactoMineR* package^21,54^. Variables used in the PCA were scaled. The Hierarchical Clustering was based on the Principal Components with Euclidean distances to calculate dissimilarity between observations, and using the largest dissimilarity between a point in the first cluster and a point in the second cluster (furthest neighbour method)^21,54^.

### Prospecting and settlement phases

To distinguish the dispersal phases, we monitored bird trajectories online using Movebank during the breeding season (March - August), after the first winter. Initially, a “settlement event” was suspected when restricted movements suggested territory establishment, detected directly on Movebank. Field observations were then conducted within 14 days to confirm settlement, looking for indications such as pairing, nesting behaviour, and territorial patrolling (individuals with restricted movement while chasing conspecifics) during several days to weeks, which indicated effective territory establishment. The “prospecting phase” then encompassed the period before the year of establishment of the first territory. For this analysis, migratory paths were identified and excluded using the Lavielle segmentation method^55^, applied to data collected outside the breeding season. The accuracy of this method was visually confirmed using Net Squared Displacement plots (following^42^ and see Figure S1). For this study, the prospecting phase thus refers to the period before the year of the first territory establishment, which can last several years (Table 1), while the settlement phase represents the entire period after establishing the first territory (following^42,56,57^).

**Table 1:** Summary of tracking data for the prospecting and the settlement phase, detailing the number of individuals (N id), individual-years (N id-yr), mean, standard deviation (SD), minimum, and maximum years tracked, along with average GPS locations per bird (N loc) and their SD.

Phase	*N* id	*N* id-yr	Mean	SD	Min	Max	*N* loc	SD loc
Prospecting phase	77	216	2.8	0.7	2	5	6093	2795
Settlement phase	77	129	1.7	0.8	1	4	5608	3271

### Step selection analyses

#### Conditional logistic regression and availability

We analysed the habitat selection along the trajectory of individual red kites using integrated step-selection analyses (iSSA). This method contrasts the locations used by the animal with the potential locations it could have accessed within a given step, using conditional logistic regression^40,41,58,59^. Available step lengths were sampled from an exponential distribution, parameterized by the observed step lengths across the entire population^41^. Turning angles for these available steps were derived from a uniform distribution, ranging from -π to π. For every observed step, we created 10 random alternative steps by using the *amt* package^53^, resulting in a dataset of 77 individuals with 5.2 million steps for the prospecting phase (*n* = 216 kite-years) and 4.8 million steps for the settlement phase (*n* = 129 kite-years, detailed sample size in Tables 1 and 2). Elevation and the proportions of CLC classes (also calculated within a 2 km buffer) were then extracted for each end-location of the observed and available steps.

**Table 2:** Count of individuals (N id) and total years tracked (N id-yr) across different natal habitats for both the prospecting and the settlement phase.

Phase	Natal Habitat	*N* id	*N* id-yr
Prospectors	Low Urban	8	22
	Low Farmland	46	134
	Medium Pasto Forest	12	31
	High Agro Mosaic	11	29
Settlers	Low Urban	8	14
	Low Farmland	46	81
	Medium Pasto Forest	12	19
	High Agro Mosaic	11	15

#### Covariates

##### Habitat dissimilarity index

**Fig. 1:**
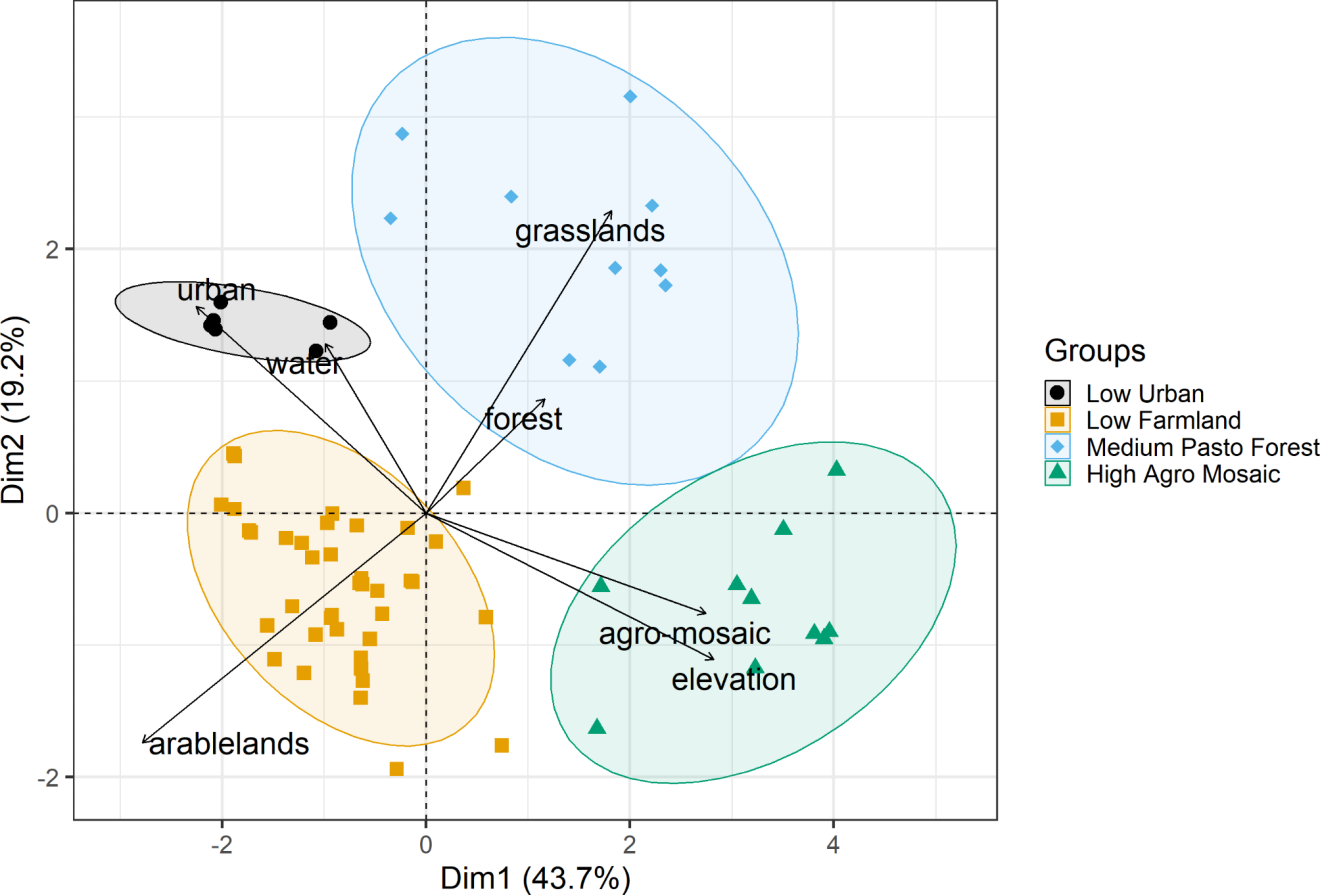
Principal Component Analysis (PCA) of habitat characteristics in the natal areas of red kites and hierarchical clustering of the individuals into natal habitat types (ellipses; *N* = 77 individuals). The two most important dimensions (PC1 and PC2) are shown. A geographical representation of these groups is provided in Figure S2, while Figure S3 details the percentage of variance explained by each PCA dimension. The largest group was the *Low Farmland* group (*n* = 46 individuals), followed by the *Medium Pasto Forest* group (*n* = 12 individuals) and the *High Agro Mosaic* (*n* = 11 individuals). The smallest group, the *Low Urban* group, comprised 8 individuals (see details in Table 2 and S1). Step locations that were both available and utilized were subsequently projected into this PCA space. The Euclidean distance (see Eq. 1) between each location and its corresponding point of natal origin was then computed, serving as an index of dissimilarity between them.

To quantify NHPI, we applied a dissimilarity index that evaluates the habitat differences between each observed step and each available step relative to the natal territory for a given individual. This was achieved by first projecting each step onto the PCA space of natal habitats. We then extracted the values of the first two principal components (PC1 and PC2) for each step’s end-location and calculated the Euclidean distance (following^26^) to each corresponding natal locations on the PCA (Fig. 1):

1 $$\:Dissimilarit{y}_{i}\:=\:\sqrt{{\left({x}_{o}-{x}_{i}\right)}^{2}+{\left({y}_{o}-{y}_{i}\right)}^{2}}$$

With x_o_ and y_0_ representing the values of PC1 and PC2 for the natal area, while x_i_ and y_i_ denote the values of PC1 and PC2 for each of the *i*-step location (both observed and available). Lower dissimilarity values therefore indicate habitats more similar to the natal habitat.

##### Distance to natal nest

To account for the tendency of individuals to select areas closer to their natal area, we also extracted the geographic distance to the natal nest (distance, in km) for each observed and available step (following^42^) and used this distance as covariate in the models. The two covariates, dissimilarity and distance, were not strongly correlated (Pearson’s correlation < 0.6).

#### Individual-year modelling of NHPI

To investigate the impact of the dissimilarity on step selection, while controlling for the distance to the natal nest, we first standardized and centred both covariates^60^. We then fitted conditional logistic regressions to each individual-year separately to specifically assess individual variation^39,41^. This analysis was conducted using the *IndRSA* package which allows for individual-iSSA and the extraction of individual-year coefficients for both covariates^39^. We hereafter refer to NHPI as the negative of the dissimilarity coefficients.

#### Inter-individual variation in NHPI

To separate population averages from inter-individual variation in NHPI coefficients, three metrics were calculated for each individual-year *i* (equations recently formulated by^39^):

2 $$\:Average=\overline{x}=\frac{1}{n}{\sum\:}_{i=1}^{n}{x}_{i}$$

NHPI average (Eq. 2) represents the population average of individual-year NHPI coefficients x_i_ with a total of *n* individual-years (*n* = 216 + 129 = 345).

3 $$\:Specialization=\:\frac{1}{n}\:\sum\:_{i=1}^{n}Abs\left({x}_{i}\right)$$

NHPI specialization (Eq. 3) represents the average of the magnitude of the individual-year NHPI coefficients x_i_ (independent of the direction), and it is strictly positive, with higher values indicating higher individual specialization. This metric can be particularly informative when the overall population average is equal to zero and/or to capture a bimodal (or more complex) pattern in a population coefficients^39^.

4 $$\:Heterogeneity=\:\sqrt{{\frac{1}{n-1}\:\sum\:_{i=1}^{n}\left({x}_{i}-\:\stackrel{-}{x}\right)}^{2}}$$

NHPI heterogeneity (Eq. 4) corresponds to the standard deviation of individual-year NHPI coefficients x_i_ with higher values indicating higher inter-individual heterogeneity.

##### Intra-individual variation in NHPI

###### NHPI change from prospecting to settlement

To quantify intra-individual variation i.e., how an individual’s NHPI coefficients change from the prospecting to settlement, we applied a consistency and reversal metrics^39^ to the dispersal phases prospecting *p* and settlement *s* (with the same notation as above):

5 $$\:Consistency=\:\frac{1}{n}\:\sum\:_{i=1}^{n}Abs({x}_{i,p}-\:{x}_{i,\:s})$$

6 $$\:Reversal=\:\frac{1}{n}\:\sum\:_{i=1}^{n}\left\{\begin{array}{c}1\:if\:sign\left({x}_{i,p}\right)\ne\:\:sign\left({x}_{i,s}\right)\:\\\:0\:if\:sign\left({x}_{i,p}\right)=\:sign\left({x}_{i,s}\right)\end{array}\right.$$

This involved re-running the step selection models with data only from the final year of prospecting with the first year of settlement per individual (*n* = 77). We specifically modelled the interaction of dispersal phase (prospecting vs. settlement) with both covariates (distance and dissimilarity). Following^39^, only the interaction terms were tested, providing a separate coefficient for each phase. These interaction coefficients were then used to compute the consistency and reversal metrics, quantifying intra-individual shifts in habitat preferences between the two phases.

NHPI consistency represents the average of the absolute differences between the coefficients of the two dispersal phases across all *n* individuals (*n* = 77, Eq. 5). Consistency is an inverse measure as values closer to zero indicate a higher consistency between prospecting and settlement (indicating minimal difference in the coefficients). Conversely, higher values indicate greater changes in the coefficients between the two phases, implying lower consistency. NHPI reversal (Eq. 6) represents the proportion of individuals showing reversal preference between the two dispersal phases, switching from positive to negative (or vice versa). It is bounded between 0 and 1 with high values indicating an increased proportion of reversal.

##### Annual variation in NHPI during prospecting

Finally, to assess interannual variation (consistency across consecutive years) for each individual during the prospecting phase (which can last several years) we customized the consistency metric by incorporating ‘year’ as an interactive factor, with both covariates, in each individual-iSSA and calculated the metric as follows:

7 $$\:Interranual\:Consistency\:=\:\frac{1}{n}\sum\:_{i=1}^{n}\left[\frac{1}{{m}_{i}-1}\sum\:_{j=1}^{{m}_{i}-1}Abs\left({x}_{i,\:j+1}-\:{x}_{i,j}\right)\right]\:\:$$

Using the same notation as before, here *m* refers to the total number of years for each individual *i.* The first summation calculates the absolute differences for each consecutive year *j* for each individual *i*. The term *m*_*i*_ – 1 normalizes these differences across individuals. The second summation thus provides an average measure of temporal NHPI consistency across *n* individuals (*n* = 77).

#### Propagation of uncertainties, models validation and residuals spatial autocorrelation

To estimate uncertainties within the five NHPI metrics (average, specialization, heterogeneity, consistency, and reversal), we applied a data augmentation method using the *IndRSA* package. This method involved the generation of 1000 simulated replicates of each individual coefficient from each covariate for each model, drawn from a normal distribution centred around the given coefficient values. The chosen standard deviation was the standard error corresponding to these coefficients (see^39^ for detailed explanations). For each set of individual simulated coefficients, we then calculated the NHPI metrics, producing 1000 values for each metric^39^. This method, similar to bootstrapping, minimizes the effects of autocorrelation by treating each individual as independent, ensuring more reliable errors estimations^41^. We then assessed model performance using k-fold cross-validation using the *indRSA* package^39^, withholding 20% of the data, assessing model fit, and repeating the process five times for each individual model. Cross-validation provided relatively high Spearman rank correlations with averages ranging from 0.63 to 0.75 indicating good model performances^61,62^. Finally, the absence of strong spatial autocorrelation in the residuals of each model was checked visually using variograms using the *geoR* package^63^.

## Results

### Principal component and clustering analyses

The first dimension of the PCA accounted for 43.7% of the variation in natal habitat type, and the second dimension accounted for 19.2% (Fig. 1 and S3). Multivariate clustering identified four natal habitat types spread along the elevation gradient (Table S1): low elevation with a relatively higher proportion of urban and village areas (hereafter, “*Low Urban*”, *n* = 8), arable lands at lower elevation (hereafter, “*Low Farmlands*”, *n* = 46), a combination of pastures and forests at medium elevation (hereafter, “*Medium Pasto Forest*”, *n* = 12), and finally a mosaic of cultivation patterns at higher elevation (hereafter, “*High Agro Mosaic*”, *n* = 11).

### Prospecting phase

**Fig. 2:**
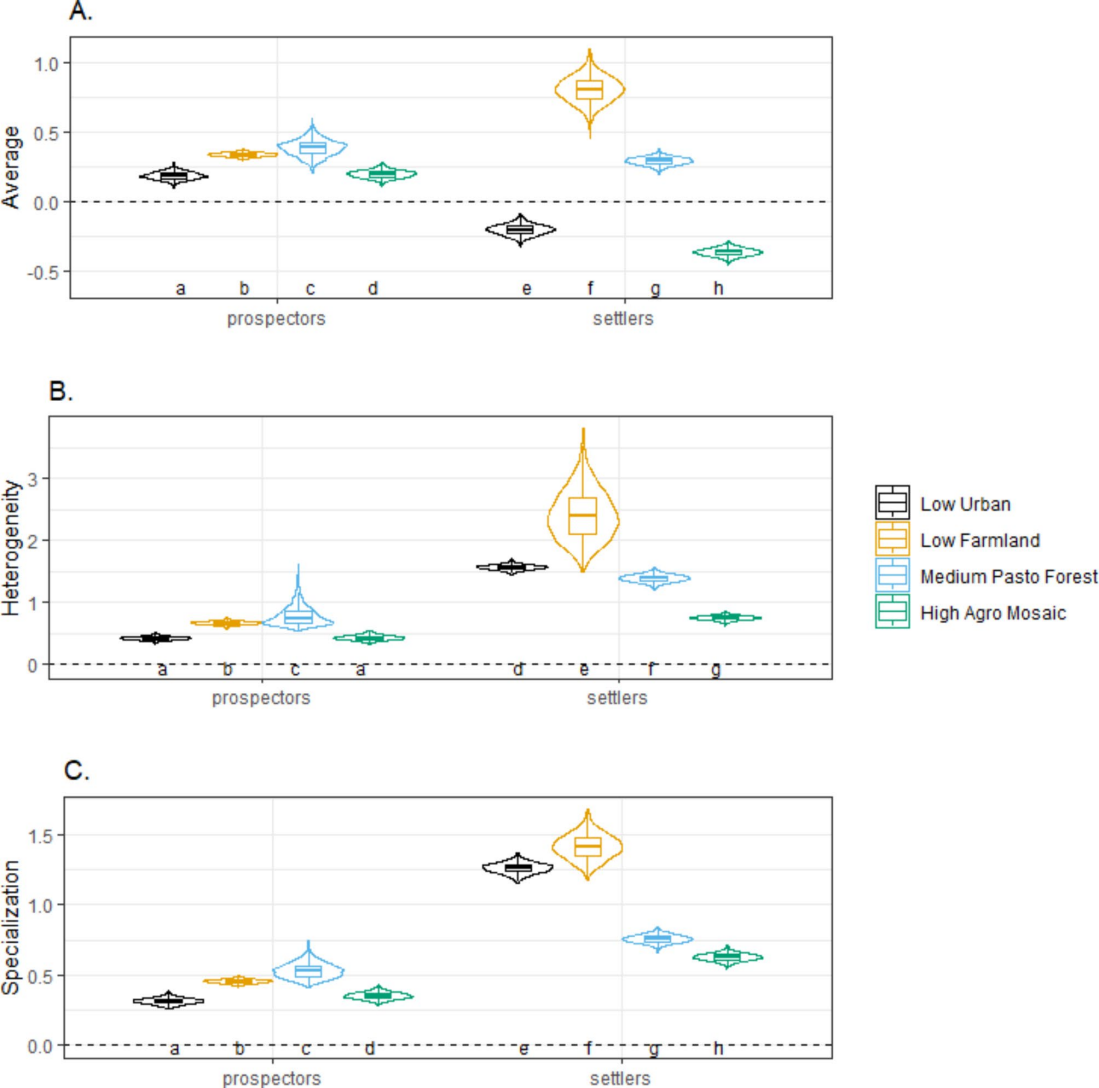
Average, heterogeneity and specialization of NHPI for the four natal habitats and for the two dispersal phases (prospecting phase and settlement phase; *N* = 77 individuals). The metrics^39^ were estimated using integrated-Step Selection Analysis (iSSA). (**A**) The Average with positive values indicates a preference for habitats more similar to natal habitats, while negative values indicate avoidance for habitats more similar to the natal ones. (**B**) Heterogeneity indicates the degree of individual variation (the standard deviation of individual coefficients). (**C**) Specialization represents the absolute magnitude of individual-year NHPI (whatever the direction of the effects). All boxplots are constructed based on simulation of 1000 replicates of individual coefficients, where tighter plots indicate lower uncertainty in the results^39^. Tukey’s HSD post hoc test letters provided where two-way ANOVA were *p* < 0.05 (different letters indicate significantly different means). Spearman rank correlations from the five-fold cross-validations were 0.63 ± 0.17 during prospecting and 0.75 ± 0.21 during settlement. For each natal habitat and dispersal phase, see Table S2 for the proportion of significant tests, and Table S5 for the proportion of the direction of the effects.

During the prospecting phase, in average, individuals from all habitat types selected habitats similar to their natal habitats (positive NHPI, Fig. 2A), but this preference was associated with high levels of NHPI heterogeneity within natal habitat types (Fig. 2B). Individuals from *Low Urban* habitats showed the weakest NHPI average (Fig. 2A) with the lowest level of NHPI heterogeneity (Fig. 2B). In contrast, individuals from *Medium Pasto Forest* habitats showed the strongest NHPI average (Fig. 2A) with the highest level of NHPI heterogeneity (Fig. 2B). The NHPI specialization values during prospecting differed from NHPI averages in all groups, thereby confirming the relatively high level of individual variation in effect direction (Fig. 2C, Table S5).

**Fig. 3:**
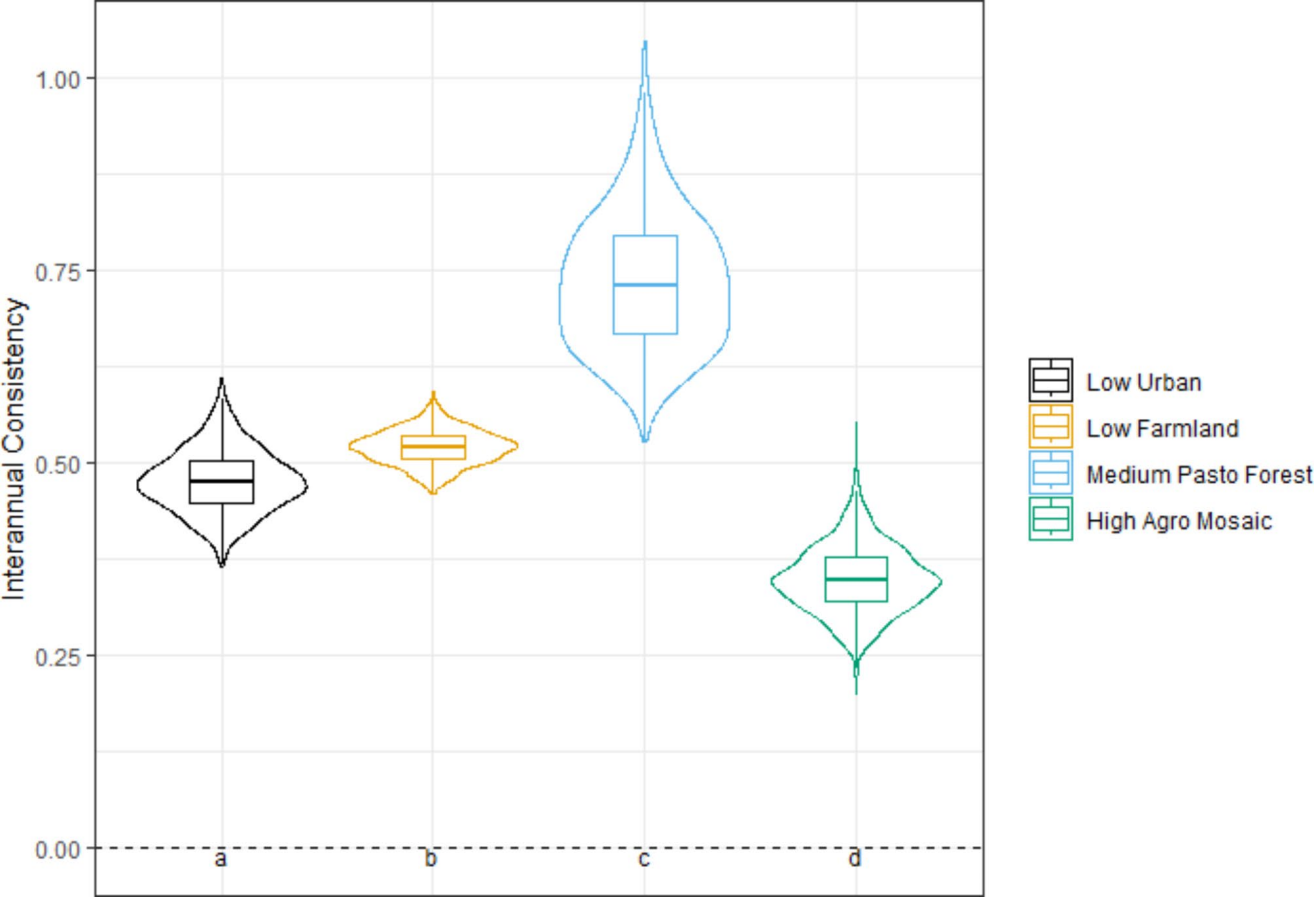
Consistency of the dissimilarity index between consecutive years of the prospecting phase for the four natal habitat types (*N* = 77 individuals). This metric (see Eq. 7) was adapted from^39^ and was estimated using integrated step selection analysis. Interannual consistency values closer to zero indicate higher consistency between years. Boxplots are constructed based on simulation of 1000 replicates of individual coefficients, where tighter plots indicate lower uncertainty in the results^39^. Tukey’s HSD post hoc test letters provided where one-way ANOVA were *p* < 0.05 (different letters indicate significantly different means). Spearman rank correlations from the five-fold cross-validations were 0.63 ± 0.19. See Table S3 for the proportion of significant tests for each natal habitat and dispersal phase.

The interannual consistency in NHPI within individuals during prospecting was relatively low, as evidenced by large values of interannual differences. The consistency metric, being of the same order of magnitude as the NHPI averages (Fig. 3), again indicated a relative high level of individual variation, here in the direction of the effects from one year to another. However, interannual consistency in NHPI also varied among habitat types. Individuals from *High Agro Mosaic* habitats exhibited the highest interannual consistency (interannual differences closer to zero; Fig. 3). Individuals from *Low Urban* and *Low Farmland* habitats showed intermediate interannual consistency, while those from *Medium Pasto Forest* habitats were the least consistent (Fig. 3).

### Settlement phase

Averages in NHPI significantly changed from the prospecting to the settlement phase, with the direction of these changes varying depending on the natal habitat. In the settlement phase, individuals from *Low Farmland* habitats showed the strongest NHPI average (Fig. 2A) but with the highest level of NHPI heterogeneity, whereas NHPI average slightly decreased for individuals from *Medium Pasto Forest* (Fig. 2B). Notably, individuals originating from *Low Urban* and *High Agro Mosaic* habitats were preferably selecting habitats dissimilar to their natal environments (negative NHPI, Fig. 2A), which was coupled with high levels of NHPI heterogeneity for *Low Urban*, but the lowest levels for *High Agro Mosaic* (Fig. 2B). Moreover, NHPI specialization values were systematically higher during settlement than during prospecting, particularly for individuals from *Low Urban* and *Low Farmland* habitats, which suggests that these individuals exhibited a stronger magnitude of NHPI (regardless of the direction; positive or negative) during the settlement than during the prospecting phase (Fig. 2C).

**Fig. 4:**
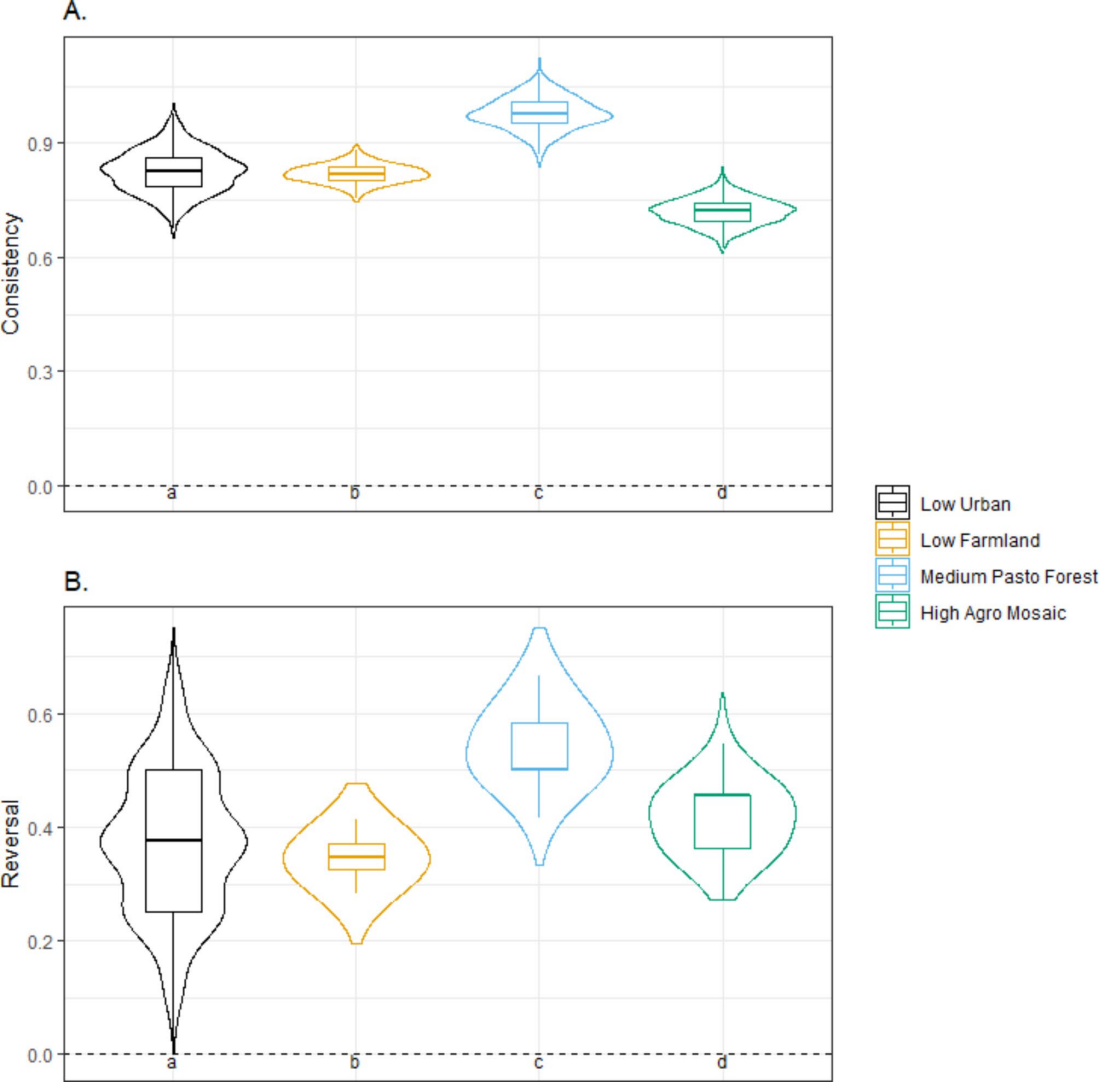
Consistency of the dissimilarity index between the last prospecting year and the first settlement year for the four natal habitat types (*N* = 77 individuals). (**A**) Consistency values closer to zero indicate higher consistency between two dispersal phases. (**B**) Reversal values represent the proportion of individuals showing reversal preference between the two dispersal phases, switching from positive to negative (or vice versa). All boxplots are constructed based on simulation of 1000 replicates of individual coefficients, where tighter plots indicate lower uncertainty in the results^39^. Tukey’s HSD post hoc test letters provided where one-way ANOVA were *p* < 0.05 (different letters indicate significantly different means). Spearman rank correlations from the five-fold cross-validations were 0.73 ± 0.18. Refer to Table S4 for the proportion of significant tests for each natal habitat and dispersal phase.

NHPI consistency values from prospecting to settlement were of the same order of magnitude as the NHPI averages (Fig. 4A), indicating that consistency was relatively low for all groups (NHPI consistency > 0). In line with this, the NHPI reversal from prospecting to settlement were relatively high for all natal habitats (between 20% and 60%), also indicating a substantial amount of intra-individual variation in the change of NHPI effects between prospecting and settlement (Fig. 4B). Nevertheless, NHPI consistency between prospecting and settlement also varied in relation to natal habitat types. Individuals from *Low Urban* and *Medium Pasto Forest* habitats were the least consistent (i.e. showed the lowest consistency value, Fig. 4A) and showed high reversal (Fig. 4B). Individuals from *High Agro Mosaic* habitats were the most consistent but also with a relatively high NHPI reversal. Lastly, individuals from the *Low Farmland* habitats, showed intermediate NHPI consistency (Fig. 4A) and reversal (Fig. 4B).

### Distances from the natal nests

In general, individuals were closer to their natal area during the settlement phase, with distances varying depending on the natal habitat type (ranging from 9 km to 54 km, Table 3). However, the effects of distance to the natal nest were negative, indicating a preference for areas closer to the natal regions during both dispersal phases (Figure S4). During the settlement phase, the observed average was less pronounced but with higher specialization and decreased consistency, suggesting that some individuals preferred larger distances (i.e. long-distance dispersers, Figure S4).

**Table 3:** Mean, median, and standard deviation (SD) of all distances to natal nests (in km) for each natal habitat and for both the prospecting and the settlement phase.

Phase	Natal habitat	Mean	Median	SD
Prospecting phase	Low urban	34.4	12	56.5
	Low farmland	47.3	6.2	90.9
	Medium pasto forest	41.1	8.5	83.3
	High agro mosaic	27.9	5.6	69.1
Settlement phase	Low urban	9.2	5.2	24.9
	Low farmland	34.0	5.5	72.3
	Medium pasto forest	16.2	3.7	34.1
	High agro mosaic	54.4	6.9	155.4

## Discussion

Our investigation into the habitat selection behaviours of juvenile red kites has provided compelling evidence of Natal Habitat Preference Induction (NHPI) during natal dispersal, although the effects were relatively weak due to substantial individual variation. Throughout the prospecting phase, most juveniles demonstrated a general trend of positive NHPI. However, from the prospecting to the settlement phase, the intensity and direction of NHPI, as well as the amount of individual variation varied depending on the natal habitat. Notably, juveniles originating from high elevation habitats or more urban areas often exhibited a shift towards avoiding their natal environments (manifesting negative NHPI) during settlement. In contrast, those from other habitats generally maintained or significantly intensified their positive NHPI upon transitioning to the settlement phase. These findings indicate an ontogenetic shift in NHPI that is influenced by the characteristics of the natal habitat, underscoring the fundamental impact of natal habitat on the development of NHPI expression and on the resulting individual variation in habitat selection behaviours.

The majority of individuals originating from all four natal habitats showed positive NHPI during prospecting, which spanned several years. This pattern is consistent with the few recent studies in the field^24,29^. Despite the need for juveniles to gather information about potential future settlement habitats after gaining independence, most of them seem to prefer habitats similar to their natal habitat, at least for satisfying their immediate needs. This inclination suggests that familiarity with specific features of the natal habitat, such as prey species, foraging areas, or roosting sites, could provide benefits not just for reproductive success but also during dispersal. Such preferences for familiar habitats may lower mortality risks and the energy expenditure associated with dispersal, potentially facilitating long-distance movements before settlement^64^.

During the prospecting phase, the occurrence of NHPI generally leads non-breeding individuals within the same population to select a range of different habitats. However, significant individual variation in NHPI expression, as indicated by the heterogeneity and specialization metrics, reveals that even within a single natal habitat, individuals exhibit different magnitudes of NHPI. This variation demonstrates diverse degrees of individual specialization in habitat use, where each individual selects habitat differently based on its natal experiences, despite belonging to the same population. This phenomenon causes the species’ broad ecological niches^42,44,65^ to divide into more specific niches at the individual level even before settlement occurs. Consequently, NHPI during dispersal is likely an important mechanism for the development of individual niches^66^. This individual behavioural specialization is then likely to lead to the known dietary and habitat specializations, also observed during the breeding season in other species^67,68^. Our findings also indicate that the degree of specialization in habitat selection increases from the prospecting to the settlement phase. Therefore, the overall pattern of habitat selection in red kites^42^ could be seen as a complex interplay of changes in specialization, ontogenetic shifts in NHPI, and the uneven distribution of specialized individuals across different natal habitats.

NHPI is generally considered to be associated with learning processes occurring from early life under parental care to post-independence phases within the natal environment, representing a form of non-genetic inheritance^12^. However, the type of natal habitat also appears to have a clear effect on the extent of NHPI exhibited during dispersal. We see two possible explanations for this pattern. Firstly, imprinting might lead individuals to develop habitat fidelity or prey specialization. This process suggests that animals not only acquire general survival skills within their natal habitats but also form strong attachments to more specific habitat features and prey types, guided by early experiences. These specialized behaviours and preferences, also indicative of NHPI, could be hypothesized to account for the observed variations in NHPI strength across different habitats. This specialization hypothesis posits that the impact of NHPI is nuanced and varies, influenced not only by unique interactions and experiences within specific natal environments but also by the transferability of these learned specializations to other habitats where similar resources are available. Hence, the extent of NHPI may differ based on the availability of familiar resources in non-natal habitats, allowing for the application of specialized knowledge beyond the natal setting^69^.

Secondly, the quality of natal habitats varies within and between habitat types. Originating from a low-quality habitat may diminish the benefits of NHPI. If unknown habitats offer more benefits than those similar to the natal ones, negative NHPI might even be observed. This adjustment of NHPI behaviour to experienced habitat quality would align with the ‘Bayesian updating’ concept, where juveniles continuously collect and revise information about habitat quality^70,71^ to enhance survival prospects during periods of high mortality risk^72^. The detection of NHPI across all habitats, its large individual variation, along with its varying direction and magnitude during the settlement phase, suggests a potential adjustment of NHPI in response to habitat quality. However, further research is necessary to clearly distinguish between the specialization and adjustment hypotheses.

NHPI changed from the prospecting to the settlement phase of dispersal, and the extent of this change was dependent on the natal habitat, associated with a high level of individual variation. In particular, the consistency and reversal metrics indicate that shifts in NHPI could also have occurred during the prospecting years, even before settlement. This ontogenetic shift in NHPI leads to two primary conclusions. Firstly, the fact that habitat selection shifts after years of prospecting implies that NHPI may offer different advantages to individuals during the prospecting and breeding stages. The transition in habitat selection varied among individuals from different natal habitats, encompassing both a marked increase in positive NHPI in the *Low Farmland* habitat and a reversal from positive to negative NHPI in *Low Urban* and in the *High Agro Mosaic* habitats. This variation can be attributed to the influence of additional resources and habitat features required for reproduction. Not every prospecting habitat is equally suited to breeding, suggesting that the breeding potential of habitats significantly influences NHPI expression upon entering the settlement phase.

Secondly, the shift in habitat selection from prospecting to settlement is unlikely to be driven by personal experience, as they have not yet established territories or reproduced. This shift in habitat selection may be influenced by observing the reproductive success of others, providing social information^73,74^, or it could be driven by inherited preferences that guide breeding habitat selection, indicating a genetic component^32,75^. This could suggest a complex interplay between genetics and social information as potential mechanisms behind this change in behaviour. The fact that NHPI at settlement can lead to ecological traps, where preferred habitats negatively impact survival and breeding, was exemplified in snail kites (*Rostrhamus sociabilis*^27^). This phenomenon suggests that intrinsic knowledge of habitat quality plays a significant role, rather than adjustments in NHPI based on social information. Overall, our results strongly suggest that similar habitats are perceived as varying in quality during different dispersal phases^8^, indicating an ontogenetic shift in the perception of habitat quality.

Evidence suggests fitness-relevant differences between habitats^76^ that align with the observed shifts in NHPI from prospecting to settlement. In Swiss lowlands, regular practices like small-scale mowing and ploughing enhance food accessibility for red kites^65^. Moreover, these arable landscapes are interspersed with forest patches that provide suitable nesting sites. Conversely, recent studies have highlighted the low quality of high elevation habitats, which are generally avoided, though more frequently explored during prospecting than settlement^42^. Additionally, breeding at higher elevations tends to start later^50^, and breeding success is lower likely due to harsher climate conditions^49^. These observations correspond closely with the marked increase in NHPI seen in *Low Farmland* habitats and the transition from positive to negative NHPI in *High Agro Mosaic* habitats upon settlement.

The *Medium Pasto Forest* habitats, positioned between forested and open landscapes, may impose constraints on resource availability during the prospecting phase. While *Medium Pasto Forest* habitats offer suitable nesting and roosting opportunities, their limited foraging possibilities likely reduce their overall suitability, especially when compared to more resource-abundant areas like *Low Farmland* habitats^49,50^. This limitation may explain the low intra-individual consistency observed in individuals from *Medium Pasto Forest* habitats, as reflected by the consistency and reversal metrics. The high variability in habitat use suggests that individuals from *Medium Pasto Forest* habitats may face trade-offs between suitable nesting and roosting resources and foraging opportunities, leading to greater fluctuations in habitat selection.

Our findings regarding urban habitats add to the growing literature on the development of urban animal populations^77,78^. Recent research demonstrated that red kites generally avoid urban areas, especially during the settlement phase^42^. However, the increasing number of sightings of red kites in urban areas, combined with the positive NHPI observed during prospecting, suggest that urban habitats are not necessarily avoided during settlement due to a lack of foraging opportunities. Instead, the avoidance may be due to a lack of suitable breeding sites or human disturbances during the settlement phase. This indicates that red kites utilizing urban environments are not forming a distinct group but rather adapting to urban resources under specific conditions^79–81^.

As illustrated by the different NPHI metrics, we showed considerable variation both between and within individuals from the same natal habitat type, which could stem from several factors. These include differences in habitat quality within the same type, variations in the learning processes that underlie NHPI, or individual differences in adjusting NHPI during prospecting^17,33^. Habitat selection is also known to have a genetic component^75,82^. To our knowledge, only one study has disentangled environmental from genetic influences on NHPI through a cross-fostering experiment, indicating that NHPI is predominantly influenced by early experiences rather than genetic factors^32^. In our study, we found NHPI to be adjusted during ontogeny, indicating the importance of plasticity in NHPI expression. Furthermore, recent research also indicates that NHPI’s impact may decrease over time, supporting the idea that individual experiences can influence NHPI expression^26^. In red kites, recent findings demonstrate that siblings from the same nest can show different patterns of range use and habitat selection based on their dominance status^83^. Therefore, it is plausible that early social dynamics in the nest, especially competitive interactions, modulate NHPI. Notably, the variability in NHPI consistency within the same habitat and the reversal of NHPI preferences from prospecting to settlement could be partially attributed to competitive differences, as less dominant individuals might struggle to secure territories in their preferred habitats^84^.

In conclusion, our study adapted a methodology to assess both inter- and intra-individual variation in NHPI, a method not commonly found in habitat selection research^38,39^. Our approach also integrated multivariate analysis, clustering algorithms, and step selection functions with a dissimilarity index, enabling the evaluation of habitat preferences across landscape gradients. By distinguishing distinct habitats and utilizing the dissimilarity index as a continuous variable, we effectively capture the dynamic adjustments individuals make between their natal and encountered environments during dispersal. Our study revealed significant individual variations in NHPI that are influenced by natal origin, landscape gradients, and ontogeny, highlighting the importance of early life experiences in movement and life-history strategies, as well as the plasticity of natal habitat preferences. Through this innovative approach, we aimed to pave the way for further investigations into how diverse natal habitats could influence habitat selection later in life.

## Electronic supplementary material

Below is the link to the electronic supplementary material.

Supplementary Material 1

## Data Availability

The GPS datasets analysed in the current study are available in Movebank (www.movebank.org), under the project named “Milvusmilvus_Milsar_SOI_final” (ID 1356790386) and "Milvusmilvus_GSM_SOI" (ID 230545451). Data and codes to reproduce the analyses are made available through the Open Repository and Archive from vogelwarte.ch (https://doi.org/10.5281/zenodo.13970545).
