# Supplementary material for: Imprinted habitat selection varies across dispersal phases in a raptor species
Source: Sci Rep. 2024 Nov 4;14:26656. doi: 10.1038/s41598-024-75815-1 (PMC11535207; doi:10.1038/s41598-024-75815-1)
Supplement: Supplementary file 1 — Supplementary Material 1 [file 41598_2024_75815_MOESM1_ESM.docx]

**Imprinted habitat selection varies across dispersal phases in a raptor species**

Florian Orgeret^1*^, Urs G. Kormann^1^, Benedetta Catitti^1^, Stephanie Witczak^1^, Valentijn S. van Bergen^1^, Patrick Scherler^1^, Martin U. Grüebler^1^

^1^ Swiss Ornithological Institute, Sempach, Switzerland. *florianorgeret@gmail.com

**Supplementary information**


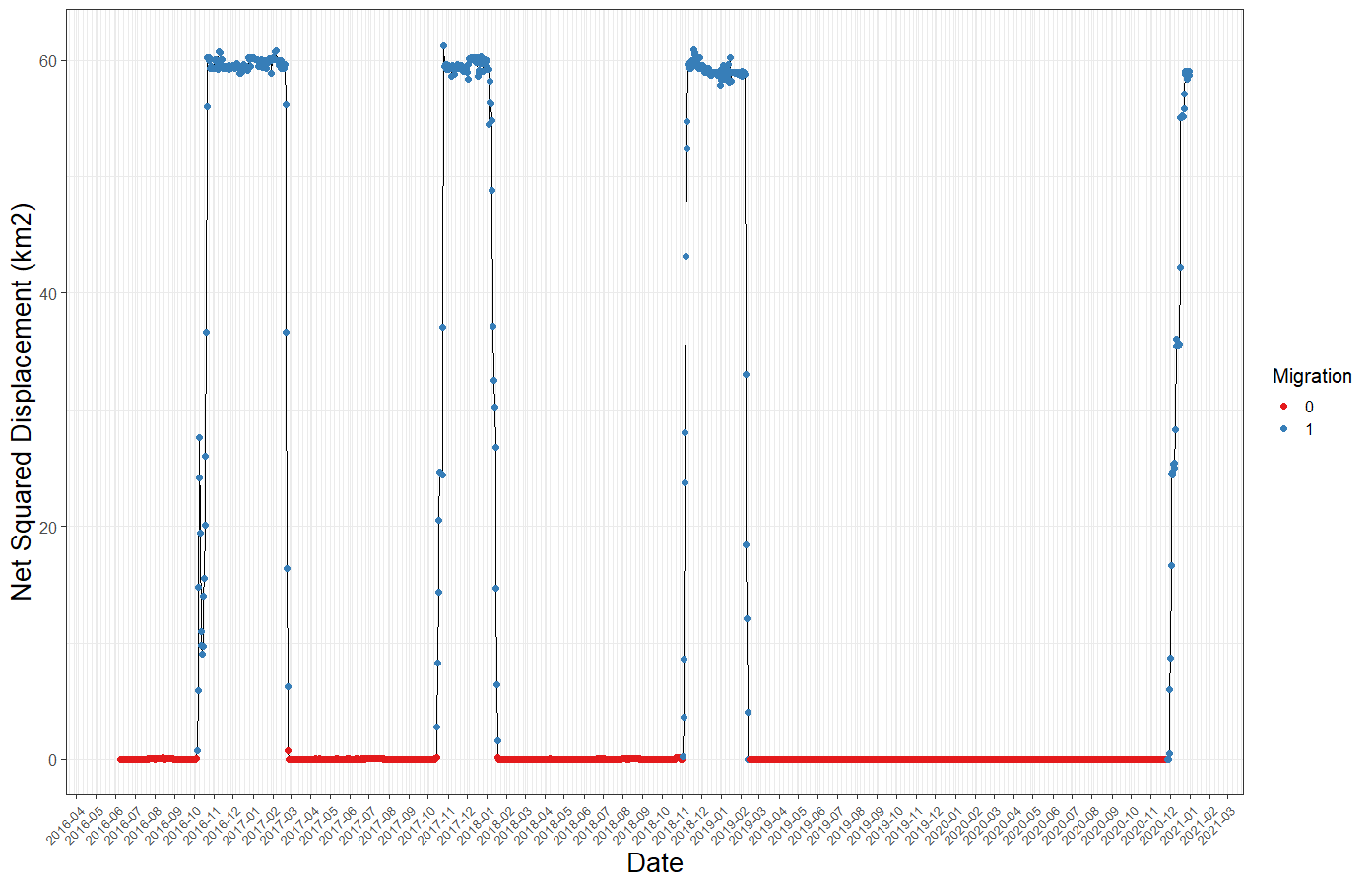


**Figure S1:** Example of a Net Squared Displacement plot, for one individual with identification of the migration phase (in blue) done using the Lavielle segmentation method. The NSD is calculated as the squared distance between the first location (first GPS localization at the natal nest) and subsequent relocations (averaged per day). This method allowed the identification of the migration dates for all individuals. Here, the individual migrated 4 times (last migration phase not complete). In this study, migration phases were excluded and only the dispersal phases were kept in the analyses.

**
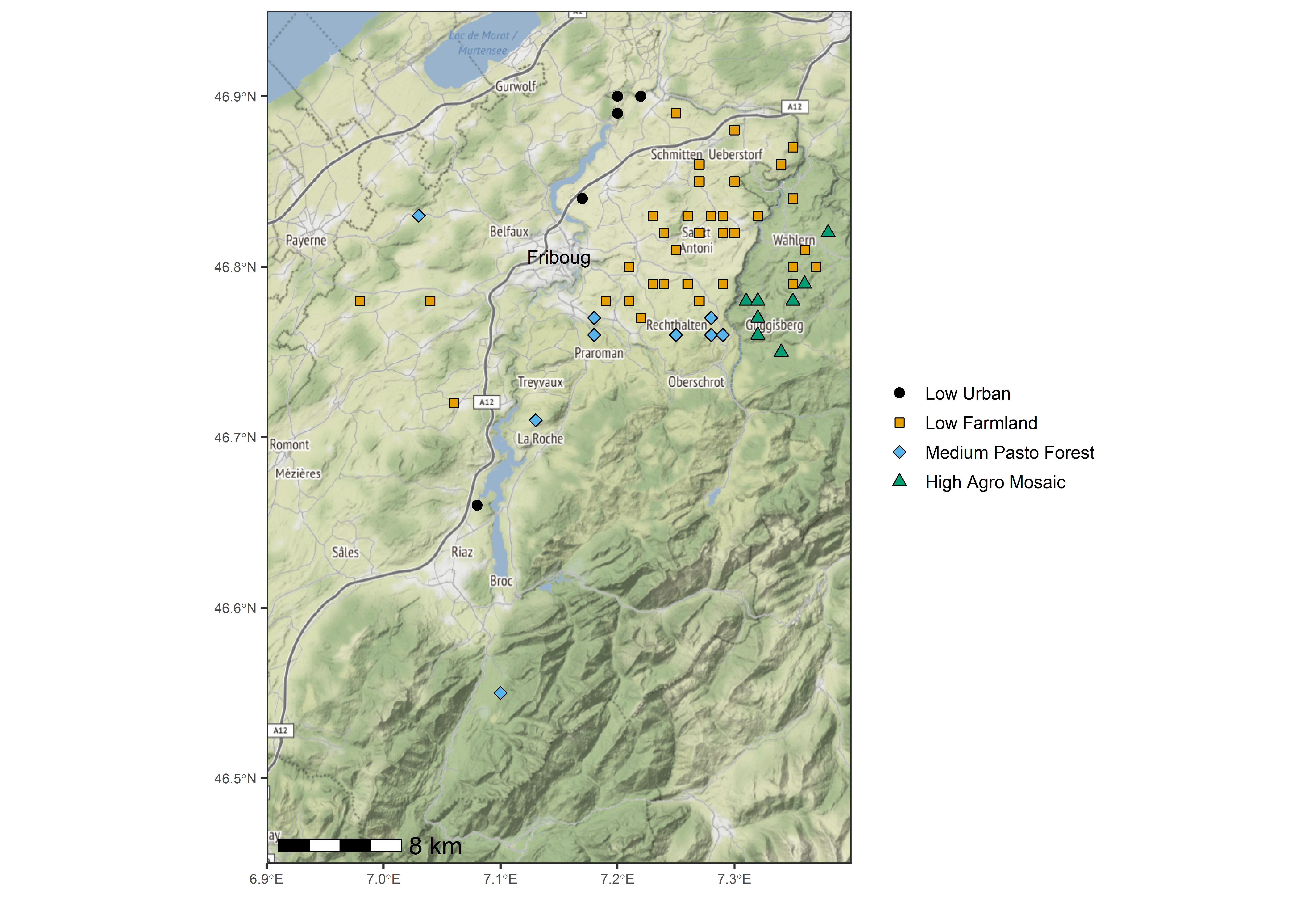
**

**Figure S2:** Map of nest locations for individual red kites, colour-coded by the distinct natal habitat type identified through hierarchical clustering based on PCA values (**Figure 1**). The clustering reveals differences in environmental characteristics, including Corine Land Cover classes and elevation, within a 2 km radius buffer around the natal nest (**Table S1**). The colour scheme represents the specific groups, reflecting variations in habitat types across the study area (background map from ©[OpenStreetMap](http://www.openstreetmap.org/copyright)).


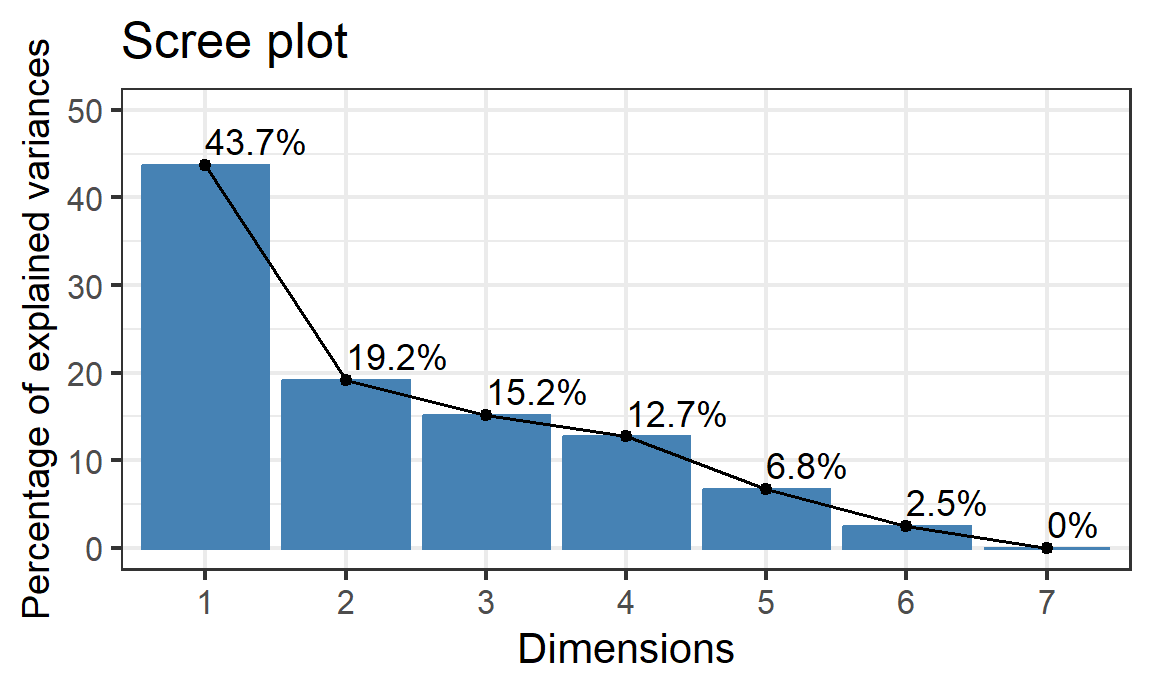


**Figure S3:** Percentage of explained deviances for all dimensions of the PCA of natal environments (**Figure 1**).

**
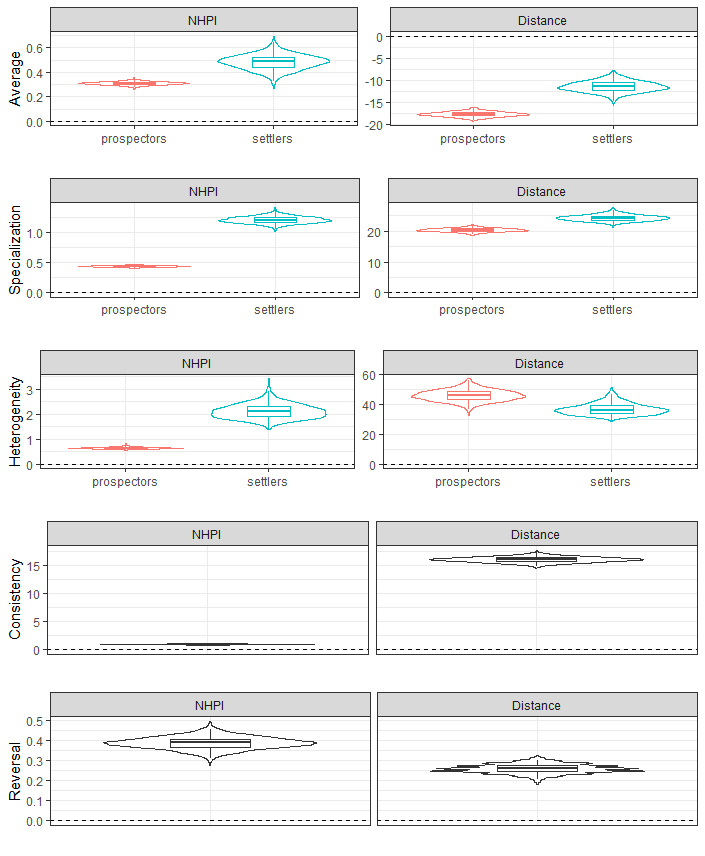
**

**Figure S4:** Violin and boxplots of individual variation in metrics for the NHPI (left panels) and for the distance to the natal nest (right panels) of 77 red kites, according to their dispersal phases (prospecting to settlement for the NHPI metrics average, specialization, and heterogeneity). Since consistency and reversal compare the two dispersal phases (prospecting and settlement) only one boxplot for each covariate is presented. These metrics^38^ were estimated using integrated step selection analysis. Boxplots are constructed based on simulation of 1000 replicates of individual coefficients, where tighter plots indicate lower uncertainty in the results^38^.

**Table S1:** Average percentages (± SD) of each environmental Corine Land Cover (CLC) class, and averages elevation (± SD), for the different natal habitat types, as separated by hierarchical clustering. Mean elevation (in meters) was treated as a continuous covariate, while Agro-mosaic, Urban, Water, Arable lands, grasslands and forest represent proportions of CLC classes within a 2 km radius buffer around the natal nest.

| **Group** | **N** | **Agro-Mosaic** | **Urban** | **Water** | **Arable lands** | **Grass**  **lands** | **Forest** | **Elevation** |
| --- | --- | --- | --- | --- | --- | --- | --- | --- |
| Low Urban | 8 | 6±3 | 11±5 | 10±7 | 54±6 | 3±3 | 17±9 | 575±64 |
| Low Farmland | 46 | 4±5 | 6±4 | 0 | 72±7 | 2±6 | 15±8 | 730±82 |
| Medium Pasto Forest | 12 | 10±9 | 7±5 | 0 | 16±10 | 46±13 | 21±6 | 795±95 |
| High Agro Mosaic | 11 | 49±13 | 0 | 0 | 17±16 | 12±6 | 22±11 | 998±88 |

| **Table S2:** Proportion of significant tests (p-values < 0.05) for dissimilarity and distance covariates, with coefficients used to calculate metrics of inter-individual variation (Average, Heterogeneity and Specialization). | | | | | |
| --- | --- | --- | --- | --- | --- |
| **phase** | **term** | **clust** | **n_sign** | **n_test** | **proportion** |
| prospecting | dissimilarity | Low Urban | 11 | 22 | 0.50 |
| prospecting | distance | Low Urban | 18 | 22 | 0.82 |
| prospecting | dissimilarity | Low Farmland | 92 | 134 | 0.69 |
| prospecting | distance | Low Farmland | 107 | 134 | 0.80 |
| prospecting | dissimilarity | Medium Pasto-Forest | 20 | 31 | 0.65 |
| prospecting | distance | Medium Pasto-Forest | 22 | 31 | 0.71 |
| prospecting | dissimilarity | High Agro-Mosaic | 19 | 29 | 0.66 |
| prospecting | distance | High Agro-Mosaic | 26 | 29 | 0.90 |
| settlement | dissimilarity | Low Urban | 12 | 14 | 0.86 |
| settlement | distance | Low Urban | 17 | 14 | 0.79 |
| settlement | dissimilarity | Low Farmland | 66 | 81 | 0.81 |
| settlement | distance | Low Farmland | 51 | 81 | 0.63 |
| settlement | dissimilarity | Medium Pasto-Forest | 15 | 19 | 0.79 |
| settlement | distance | Medium Pasto-Forest | 17 | 19 | 0.89 |
| settlement | dissimilarity | High Agro-Mosaic | 11 | 15 | 0.73 |
| settlement | distance | High Agro-Mosaic | 10 | 15 | 0.67 |

| **Table S3:** Proportion of significant tests (p-values < 0.05) for dissimilarity and distance covariates, with coefficients used to calculate metric of Interannual Consistency during the prospecting phase. | | | | |
| --- | --- | --- | --- | --- |
| **term** | **clust** | **n_sign** | **n_test** | **proportion** |
| dissimilarity:yr | Low Urban | 11 | 22 | 0.50 |
| distance:yr | Low Urban | 18 | 22 | 0.82 |
| dissimilarity:yr | Low Farmland | 93 | 134 | 0.69 |
| distance:yr | Low Farmland | 108 | 134 | 0.81 |
| dissimilarity:yr | Medium Pasto Forest | 20 | 31 | 0.65 |
| distance:yr | Medium Pasto Forest | 22 | 31 | 0.71 |
| dissimilarity:yr | High Agro Mosaic | 19 | 29 | 0.66 |
| distance:yr | High Agro Mosaic | 25 | 29 | 0.86 |

| **Table S4:** Proportion of significant tests (p-values < 0.05) for dissimilarity and distance covariates, with coefficients used to calculate metric of inter-annual variation (Consistency and Reversal). | | | | |
| --- | --- | --- | --- | --- |
| **term** | **clust** | **n_sign** | **n_test** | **proportion** |
| settlers:dissimilarity | Low Urban | 7 | 8 | 0.88 |
| dissimilarity:prospectors | Low Urban | 2 | 8 | 0.25 |
| distance:prospectors | Low Urban | 7 | 8 | 0.88 |
| distance:settlers | Low Urban | 7 | 8 | 0.88 |
| settlers:dissimilarity | Low Farmland | 39 | 46 | 0.85 |
| dissimilarity:prospectors | Low Farmland | 29 | 46 | 0.63 |
| distance:prospectors | Low Farmland | 38 | 46 | 0.83 |
| distance:settlers | Low Farmland | 34 | 46 | 0.74 |
| settlers:dissimilarity | Medium Pasto- Forest | 8 | 12 | 0.67 |
| dissimilarity:prospectors | Medium Pasto-Forest | 8 | 12 | 0.67 |
| distance:prospectors | Medium Pasto-Forest | 11 | 12 | 0.92 |
| distance:settlers | Medium Pasto-Forest | 11 | 12 | 0.92 |
| settlers:dissimilarity | High Agro Mosaic | 9 | 11 | 0.82 |
| dissimilarity:prospectors | High Agro Mosaic | 9 | 11 | 0.82 |
| distance:prospectors | High Agro Mosaic | 10 | 11 | 0.91 |
| distance:settlers | High Agro Mosaic | 8 | 11 | 0.73 |

| **Table S5**: Proportion of NHPI effects direction per natal habitat and dispersal phase for each individual-year model. | | | | |
| --- | --- | --- | --- | --- |
| **phase** | **clust** | **effect_direction** | **n_id_yr** | **proportion** |
| prospecting | Low Urban | negative | 9 | 0.41 |
| prospecting | Low Urban | positive | 13 | 0.59 |
| prospecting | Low Farmland | negative | 36 | 0.27 |
| prospecting | Low Farmland | positive | 98 | 0.73 |
| prospecting | Medium Pasto Forest | negative | 12 | 0.39 |
| prospecting | Medium Pasto Forest | positive | 19 | 0.61 |
| prospecting | High Agro Mosaic | negative | 6 | 0.21 |
| prospecting | High Agro Mosaic | positive | 23 | 0.79 |
| settlement | Low Urban | negative | 8 | 0.57 |
| settlement | Low Urban | positive | 6 | 0.43 |
| settlement | Low Farmland | negative | 29 | 0.36 |
| settlement | Low Farmland | positive | 52 | 0.64 |
| settlement | Medium Pasto Forest | negative | 9 | 0.47 |
| settlement | Medium Pasto Forest | positive | 10 | 0.53 |
| settlement | High Agro Mosaic | negative | 12 | 0.80 |
| settlement | High Agro Mosaic | positive | 3 | 0.20 |
